# Spectrum of Mutations in *PTPN11* in Russian Cohort

**DOI:** 10.3390/genes15030345

**Published:** 2024-03-07

**Authors:** Anna Orlova, Daria Guseva, Nina Demina, Aleksander Polyakov, Oksana Ryzhkova

**Affiliations:** 1 SRC «Genome», Research Centre for Medical Genetics, 115522 Moscow, Russia; ryzhkova@dnalab.ru; 2 Counselling Unit, Research Centre for Medical Genetics, 115522 Moscow, Russia; guseva@med-gen.ru (D.G.); ndemina47@mail.ru (N.D.); 3 DNA-Diagnostics Laboratory, Research Centre for Medical Genetics, 115522 Moscow, Russia; apol@dnalab.ru

**Keywords:** Noonan syndrome, *PTPN11* gene, population study, LEOPARD syndrome, NSML

## Abstract

Noonan syndrome is a group of diseases with a similar clinical picture, consisting of 16 diseases caused by mutations in 15 genes. According to the literature, approximately half of all cases are attributed to Noonan syndrome type 1, NSML, caused by mutations in the *PTPN11* gene. We analyzed 456 unrelated probands using a gene panel NGS, and in 206 cases, the cause of the disease was identified. Approximately half of the cases (107) were caused by variants in the *PTPN11* gene, including three previously undescribed variants, one of which was classified as VOUS, and the other two as LP causative complex alleles. Frequent variants of the *PTPN11* gene characteristics for Russian patients were identified, accounting for more than 38% (c.922A>G p.Asn308Asp, c.417G>C p.Glu139Asp, c.1403C>T p.Thr468Met) of all cases with mutations in the *PTPN11* gene. A comparative characterization of frequent variants of the *PTPN11* gene in different populations is shown. The most common features of Noonan syndrome in the studied sample were facial dysmorphisms and cardiovascular system abnormalities. A lower representation of patients with growth delay was observed compared to previously described samples.

## 1. Introduction

Noonan Syndrome (NS) is a disorder characterized by genetic heterogeneity but sharing a classical phenotypic picture, including facial dysmorphisms, a wide range of congenital heart defects, and short stature. Additional features may include skeletal deformities, neck with skin folds, cryptorchidism, and developmental disorders of male reproductive system. In some cases, intellectual disabilities may also be present [1,2,3,4,5,6,7,8,9,10]. According to the literature data, up to 50% of diagnosed and molecularly confirmed cases of the syndrome are attributed to mutations in the *PTPN11* gene [11,12].

*PTPN11* encodes the non-receptor protein tyrosine phosphatase 2 (SH2) with a Src homology region, containing protein tyrosine phosphatase 2 or SHP2 protein. The structure and function of this protein, which is a part of the RAS-MAPK (RAS mitogen-activated protein kinase) signaling pathway, are highly conserved from invertebrates to mammals [13,14].

The RAS-MAPK signaling pathway plays an important role in regulating cell proliferation, differentiation, migration, and apoptosis during embryonic and postnatal development [15]. Inherited mutations in genes encoding proteins of this pathway lead to diseases characterized by constant activation of the RAS-MAPK pathway, enhanced and unregulated protein expression, and, consequently, the entire pathway. The regulation of cell proliferation is important not only during the process of organism development but also in oncogenic transformation and carcinogenesis associated with activating mutations in RAS-MAPK genes [16].

Noonan Syndrome and Noonan Syndrome with multiple lentigines (NS and NSML, MIM*151100) result from gain-of-function mutations in *PTPN11* [17]). Loss-of-function mutations, in contrast, lead to skeletal anomalies and metachondromatosis (MC; MIM#156250).

NSML (Noonan Syndrome with multiple lentigines) is similar to NS but has a characteristic feature: multiple lentigines throughout the body. Previously, this syndrome was called LEOPARD, and this name revealed its main phenotypic manifestations: Lentigines, Electrocardiographic conduction abnormalities, Ocular hypertelorism, Pulmonic stenosis, Abnormal genitalia, Retardation of growth, and sensorineural Deafness [18]. Previously, mutations leading to NSML syndrome were thought to be associated with a decrease or loss of protein activity, in contrast to Noonan syndrome with hyperactivity and an inability to reduce or stop kinase activity [15]. However, further study of the spectrum of mutations leading to these conditions showed that NS and NSML are allelic disorders. There is some tendency indicating that NSML is more likely to occur with mutations in exons 7 and 12, but manifestations can also occur with mutations in other exons [19].

## 2. Materials and Methods

This study analyzed DNA of 456 unrelated Noonan and Noonan-phenotype-like syndrome patients. For most patients, clinical examination data and diagnoses were available.

Most patients were diagnosed at the Research and Counseling Department of the Research Centre for Medical Genetics (RCMG), including patients that were referred from the Genetic Counseling Departments of Russia.

The study was approved by the local ethics committee of the Federal State Budgetary Institution “Research Centre for Medical Genetics” (the approval number 4/1 from 19 April 2021) and all the patients gave written informed consent. All experiments were performed in accordance with the institutional guidelines.

For the current research, the Noonan syndrome Panel of target genes was developed. It comprises the following NS associated genes: *NRAS*, *RIT1*, *SHOC2*, *CBL*, *PTPN11*, *HRAS*, *KRAS*, *A2ML1*, *SOS2*, *SPRED1*, *MAP2K1*, *MAP2K2*, *PPP1R13L*, *SOS1*, *WDR35*, *LZTR1*, *RAF1*, *RASA2*, *IFT80*, *NEK1*, *RASA1*, *BRAF*, *NF1*. Next-generation sequencing of patient’s DNA was performed by an Ion S5 next-generation sequencer (Thermo Fisher Scientific, Waltham, MA, USA) with an Ion AmpliSeq™ Library Kit 2.0 (Thermo Fisher Scientific, Waltham, MA, USA) according to the manufacturer’s protocol. Patient’s DNA samples were prepared using ultra rapid multiplex PCR technology combined with subsequent sequencing (AmpliSeq™). The coverage of the *PTPN11* (RefSeq NM_002834.5) gene (exons and intron-exon junctions) was 98.39%, 1 exon was not covered. Less than 1% of the coding sequence of the gene consists of homopolymeric regions.

The variants identified were named using the nomenclature provided on the website http://varnomen.hgvs.org/recommendations/DNA (accessed on 31 May 2023) version 2.15.11 and version 20.05.

The sequencing data processing was performed using the standard automated algorithm offered by ThermoFisher Scientific (Torrent Suite™) and the NGSdata software v.2022.1 developed by N.S. Beskorovainy. The NGSdata software was registered under the number 2021614055 in 2021.

For assessing the population frequencies of the identified variants, samples from the Genome Aggregation Database (gnomAD v.2.1.1) and a sample of 2600 patients from the Russian Federation with other clinical diagnoses, not showing symptoms of Noonan syndrome (NS) or NSML, were used. [Database of nucleotide sequence variants “RuExAc”//Accessed via online service “NGSData”. URL: http://ngs-data.ru/vcfdb/ (accessed on 11 July 2023)].

To evaluate the clinical relevance of the identified variants, databases such as OMIM, HGMD^®^ Professional (database of pathogenic variants), and literary data were used.

For validation and confirmation of the presence of mutations, Sanger sequencing method with forward and reverse primers was applied. The sequencing was conducted using the protocol of the manufacturer on the ABI Prism 3100 instrument (Applied Biosystems (Thermo Fisher Scientific, Waltham, MA, USA)). Conditions of reactions and primer sequences can be provided upon request.

Statistical analysis was carried out by using the two tailed Fisher’s exact probability test for 2 × 2 contingence-table analysis. A *p* value < 0.050 was considered statistically significant.

## 3. Results

From the 456 patients referred for panel testing with diagnoses of Noonan syndrome (NS) and related conditions, possible causal mutations (P, LP, VOUS) were identified in 206 cases, 21 of which were classified as VOUS (variant of uncertain significance).

The proportion of patients with mutations in the *PTPN11* gene was 53.1% (107 cases out of the 206 identified cases). Out of these cases, there were 106 cases with P and LP.

Among patients with identified causative mutations in the *PTPN11* gene, 84.8% (89 cases) were diagnosed with Noonan syndrome (NS), and 15.2% (16 cases) had NSML.

**Figure 1:**
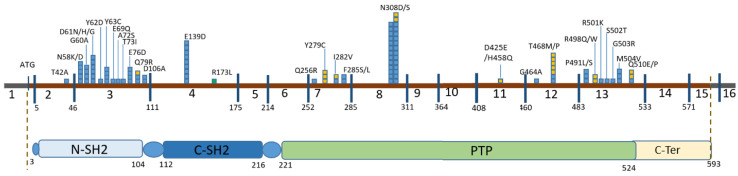
Distribution of identified pathogenic and likely pathogenic variants in the *PTPN11* gene (NM_002834). The main functional domains of the SHP2 protein are indicated at the bottom. The coding sequence is represented by a brown line, divided into exons. Blue squares represent probands with identified mutations and a phenotype of Noonan syndrome (NS), yellow squares represent probands with Café au lait patches, and green squares represent variants classified as VOUS (variant of uncertain significance).

The spectrum of identified mutations is presented in Table 1 and Figure 1.

A third of all cases were attributed to two common mutations: the most frequent variant c.922A>G (p.Asn308Asp), which occurred in 23 out of 105 cases (21.9%), and the variant c.417G>C (p.Glu139Asp), which was found in 11 cases (10.5%).

The distribution of identified variants in the gene is shown in Figure 1. The main array of identified mutations was evenly represented in exons 3 and 8 (33 and 28 cases out of 105, respectively). However, in exon 8, the variants c.922A>G (p.Asn308Asp) and c.923A>G (p.Asn308Ser) were nearly always repeated in almost all cases. In contrast, exon 3 displayed the highest diversity of identified variants.

All pathogenic variants identified in our cohort of patients with Noonan syndrome and Noonan-like conditions are previously registered missense changes (the variant c.1493G>A (p.Arg498Gln) is not registered in the ClinVar database RCV002471444.1) and are located in positions that are conserved among orthologous *PTPN11* genes in vertebrates. The majority of the identified variants in our cohort are located in exons 3 and 8, constituting 58% of pathogenic *PTPN11* variants (Figure 1).

Three novel variants were also identified and initially classified as variants of uncertain clinical significance (VOUS): c.518G>T (p.Arg173Leu), c.1275C>G (p.Asp425Glu), and c.1374C>G (p.His458Gln). It is worth noting that the p.Arg173Leu variant corresponds to a previously described variant c.518G>C (p.Arg173Pro) [20], but functional studies were not performed for the variant we detected, and its frequency in the population (0.001591%) does not contradict the prevalence of the disease (GnomAD exomes allele count = 4 is less than 5 for gene *PTPN11*). Meta In silico Predictors MetaRNN, REVEL, BayesDel addAF evaluate this variant as Pathogenic Supporting/Moderate. This variant was not found in the sample of patients from Russia with other clinical diagnoses. Since obtaining biological material and examination results from the proband’s parents was not possible, this variant remains classified as VOUS.

For variants c.1275C>G (p.Asp425Glu) and c.1374C>G (p.His458Gln), a family analysis was conducted, confirming the relationship between the proband and parents and establishing the de novo origin of the identified variants. Additionally, long-read sequencing data revealed cis-positions for these variants. The prediction programs were used to evaluate these variants separately. Meta In silico Predictors MetaRNN, REVEL, BayesDel addAF evaluate this variant as Pathogenic Strong/Moderate for each variant. Variants c.1275C>G (p.Asp425Glu) and c.1374C>G (p.His458Gln) were not detected in the GnomAD database, and they were not found in the sample of patients from Russia with other clinical diagnoses. Probably, in this case, both variants are significant as part of a complex allele for clinical manifestation. Based on the cumulative data, these variants were reclassified as likely pathogenic.

For 102 probands (63 males, 39 females) with identified causative variants and a guiding diagnosis of Noonan syndrome, NSML, or growth delay, examination data were obtained. Among these probands, 78 (76.5%) had congenital heart defects (pulmonary artery stenosis (54 cases), obstructive hypertrophic cardiomyopathy (4 cases), ventricular and valvular structural abnormalities (16 cases), atrial septal defect (10 cases), cardiomyopathy (4 cases), and other symptoms occurring in individual cases). In 48 cases (47%), short stature (<10th percentile) or delays in physical development/growth were noted, and in 35 cases (34.3% of cases with described clinical data), developmental delays (speech, psychomotor) were identified. Among probands with available clinical data and mutations in *PTPN11*, 89 (87.2%) exhibited facial dysmorphism and characteristic phenotypic features. Among the 63 males, 27 (41.5%) had cryptorchidism. Twenty probands had reduced vision (myopia) or astigmatism. Congenital kidney defects were detected in 18 probands. Hearing impairment was established in 4 cases. In 8 other cases, instrumental diagnostics of hearing impairment was not performed, but examination data suggest its possible presence.

In 16 patients (from the sample with identified mutations), the symptom complex included various spots (multiple lentigines, Café au lait patches), allowing for the diagnosis of NSML. One proband had a variant detected in exon 3 (p.Gln79Arg), and two had variants in exon 8 (p.Asn308Asp and p.Asn308Ser). Four probands had variants identified in exon 7 (p.Tyr279Cys, p.Ile282Val), and another four had variants in exon 13 (p.Arg498Trp, p.Arg498Gln, p.Gln510Glu, p.Gln510Pro). In exon 12, variants p.Thr468Met/Pro were identified in five patients. All identified variants have been previously described in patients with NSML and Noonan syndrome. Clinical manifestations in the investigated cohort are described in Table 2. It is worth noting that in the investigated sample, variants p.Tyr279Cys, p.Thr468Pro, p.Arg498Trp, p.Arg498Gln, and p.Gln510Pro were found only in patients with NSML, while other variants were found in both phenotypes.

The symptoms of probands and their correlation with the genotype are presented in the Supplementary Table S1.

## 4. Discussion

In the sample from Russia, approximately half of the patients (51.4%) with identified pathogenic and likely pathogenic variants (P/LP) had mutations in the *PTPN11* gene. According to literature data, the detectability of *PTPN11* variants relative to all identified variants in the cohort with Noonan syndrome (NS) varies widely and ranges from 20% to 60% with a median of 36% [21,22,23,24,25,26,27,28,29,30,31,32,33,34,35,36,37,38,39,40].

However, when comparing the proportions of NS cases solely caused by mutations in *PTPN11* among all confirmed cases, significant differences (*p* < 0.05) were observed only in the samples from Spain [21] and the United States [22], where the proportion of *PTPN11* mutations was significantly higher.

**Figure 2:**
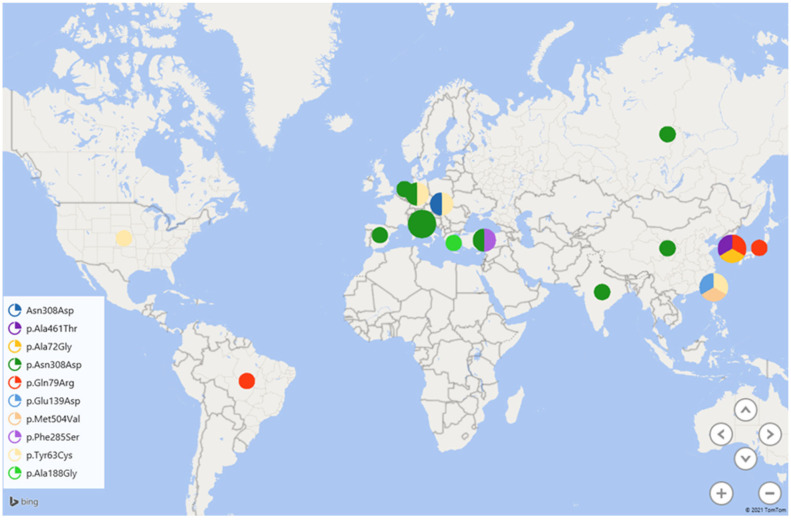
Distribution of the most commonly occurring pathogenic variants worldwide based on publications.

This increase in *PTPN11* mutations could be explained by population-specific characteristics and gene drift, as well as limitations of the applied analysis methods (as seen in the case of Spain). Since only six genes (*PTPN11*, *SOS1*, *RAF1*, *BRAF*, *KRAS*, and *HRAS*) were studied, mutations in some chromosomal regions might not have been detected, leading to an overestimation of the *PTPN11* proportion. Additionally, while the p.Asn308Asp mutation is a major mutation found in both Russia and other countries (China, India, Italy, Spain, and the Netherlands), the p.Tyr63Cys mutation is predominant among patients in the United States. Comparisons with publications that used Sanger sequencing of individual genes, gene regions, or 2–3 genes with similar phenotypic presentations were not conducted (Figure 2, Table 2).

For Germany, data from different authors differ: while Zenker et al. (2004) [23] identified p.Asn308Asp as the most common variant, Musante et al. (2003) [24] described p.Tyr63Cys as such. The latter is also found in Taiwan, the United States, and among residents of central Europe. In the Russian sample, the p.Tyr63Cys variant was identified only once. The second most frequent variant, c.417G>C (p.Glu139Asp), detected in 10.5% of chromosomes with mutations in Russian patients, is one of the common variants in Taiwan. Another frequent substitution found in 4.8% of chromosomes in Russia is an alternative variant of the most common mutation at position 308 (p.Asp308Ser). This variant is also described in other countries but is not considered major anywhere. Thus, the Asn308 variant is the cause of the disease in more than a quarter of cases in Russia (28 out of 105).

Almost all studies conducted to date on NS mutation testing have attempted to establish a correlation between genotype and phenotype. However, the most consistent feature among all of them is that there is considerable phenotypic variability even among patients with the same pathogenic variant [41].

Table 2 provides data from publications in which the occurrence of the main features of the syndrome is noted, as well as the most common variants in the sample.

The median distribution of growth delay in patients among publications is 71% (with a range of values from 0 to 100% of probands). Researchers attribute this, in part, to the fact that the majority of patients were young children examined before completing sexual maturation [42]. In the Russian patient cohort, growth delay is noted in 47% of probands.

The median distribution of probands diagnosed with developmental delay in mental development among publications is 34% (ranging from 0 probands in the Indian sample to 78% in the Chinese cohort). This variability may be due to differences in the approach to assessing mental development, the criteria used in each country, and the scales used for such diagnosis. In the Russian sample, developmental delay is reported in 34.3%. No significant differences were found for the other compared parameters.

**Table 2:** Comparison of data on major pathogenic variants in *PTPN11* and their association with phenotypic features in different populations. For publications using NGS methods (clinical exome or gene panel) and Sanger sequencing analysis of multiple genes, Fisher’s statistical analysis was used to determine the number of mutations found in *PTPN11* out of the total number of mutations found. NR—no data available.

Population/ Country Conducting the Study	Number of Samples Analyzed	Number of Variants Found, Method of Analysis	Probands with *PTPN11* (% of Variants Detected)/Number of Probands for Which a Clinic Was Available	P (Fischer)	Most Common Variant (%/Absolute Number of Person)	Delayed Growth (%/Absolute Number of Person)	P (Fischer)	Heart Defects (%/Absolute Number of Person)	P (Fischer)	Developmental Delay (%/Absolute Number of Person)	P (Fischer)
Russia (this publication)	308 (panel 23 genes)	206 (NGS)	105 (51%)/102		p.Asn308Asp (21.9%/23)	61.7%/63		77.4%/79		34.3%/35	
China [26]	NR Inherited Disease Panel (containing 2742 genes) sequencing	103 (NGS)	50 (48.5%)/50	0.688	p.Asn308Asp (28.0%/14)	74%/37	0.302	82%/41	0.181	78%/39	0.009
Italy [27]	80 (panel 11 genes)	37 (NGS)	22 (59.5%)/22	0.457	p.Asn308Asp (18.2%/4)	NR		NR		NR	
USA [22]	1254 (845 prenatal diagnostic + 409 postnatal) (panel 9 genes)	145 (NGS)	96 (66.2%) Postnatal: 63 *PTPN11* (72.4%), other 24: *SOS1* (8.0%), *RAF1* (5.7%), *BRAF* (4.6%), *SHOC2* (3.4%), *MAP2K1* (2.3%), *KRAS* (2.3%), and *HRAS* (1.1%). Prenatal: *PTPN11* 37.8% (28) *SOS1* 27% (20) 9.5% (7) for *MAP2K2*, 8.1% (6) for *RAF1*, 6.8% (5) for *BRAF*, 4.1% (3) for *HRAS*, 2.7% (2) for *KRAS* and *SHOC2* each, and only 1.4% (1) for *MAP2K1*	0.024	p.Tyr63Cys (13.5%/13)	NR		NR		NR	
Central European (Slovakia, Slovenia, Austria, Hungary, Czech Republic) [28]	51 (panel 13 genes)	35 (NGS)	22 (62.8%)/22	0.336	p.Tyr63Cys (18.2%/4) Asn308Asp (13.6%/3)	77.2%/17	0.296	63.6%/14	0.79	40.9%/9	0.332
Korea [29]	59 (Sanger, 4 genes (*PTPN11*, *SOS1*, *KRAS*, and *RAF1*))	30	16 (53.3%)/16	1	p. Ala72Gly (2/-), p.Gln79Arg (2/-), p.Ala461Thr (2/-)	56.2%/9	0.577	68.75%/11	1	25%/4	0.052
Türkiye [30]	31 (Sanger, 6 genes (*PTPN11*, *SOS1*, *KRAS*, *RAF1*, *SHOC2*, *NRAS* and *CBL*))	11	7 (63.6%)/7	0.54	p.Phe285Ser (2/-)	71.4%/5	1	71.4%/5	1	57.1%/4	1
Italy [31]	40 (Sanger, 3 genes (*PTPN11*, *KRAS*, *SOS1*))	15	14 (93.3%)/14	0.044	-	100%/14	0.007	100%/14	0.015	NR	
Spain [21]	643 (Sanger, 6 genes (*PTPN11*, *SOS1*, *RAF1*, *BRAF*, *KRAS* and *HRAS*))	230	172 (74.8%) 172 *PTPN11*+, 14 *SOS1*+, 9 *RAF1*+, 5 *BRAF*+	<0.001	p.Asn308Asp (27.3%/47)	NR		69.7%/120	1	NR	
India [25]	363 (*PTPN11*)	107 (Sanger)	107/107	-	p.Asn308Asp (11.2%/12)	43/40.2%	0.005	62.6%/7	0.495	0	<0.001
Germany [23]	57 (*PTPN11*)	34 (Sanger)	34/34	-	p.Asn308Asp (17.6%/6	28/82.3%	0.096	41.2%/14	0.015	53%/18	1
Japan [32]	45 (*PTPN11*)	18 (Sanger)	18/18	-	p.Gln79Arg (16.7%/3)	0	<0.001	83.3%/15	0.366	NR	
Italy [33]	425 (*PTPN11*)	204 (Sanger)	204/116	-	p.Asn308Asp (19.6%/40)	NR	-	NR		NR	
Italy [34]	84 (*PTPN11*)	34 (Sanger)	34/34	-	p.Asn308Asp (17.6%/6)	64.7%/22	1	58.8%/20	0.369	14.7%/5	<0.001
Germany [24]	96 (*PTPN11*)	32 (Sanger)	32/32	-	p.Tyr63Cys (28.1%/9)	43.75%/14	0.079	65.6%/21	0.815	43.75%/14	0.39
Netherlands [35]	170 (*PTPN11*)	76 (Sanger)	76/76	-	p.Asn308Asp (21.1%/16)	54%/41	0.291	81.6%/62	0.104	36.8%/28	0.079
Taiwan [36]	34 (*PTPN11*)	13 (Sanger)	13/13	-	p. Tyr63Cys (2/-), p.Glu139Asp (2/-), p.Met504Val (2/-)	84.6%/11	0.199	69.2%/9	1	84.6%/11	0.06
Greece [37]	80 (*PTPN11*)	17 (Sanger)	17/17	-	p.Ala188Gly (29.4%/5)	76.5%/13	0.393	64.7%/11	0.773	64.7%/11	0.58
Egypt [38]	NR (*PTPN11*)	21 (Sanger)	21/21	-	NR	71.4%/15	0.6	71.4%/15	1	52.4%/11	1
Brazil [39]	50 (*PTPN11*)	21 (Sanger)	21/21	-	p.Gln79Arg (3/14.2%)	95.2%/20	0.005	90.5%/19	0.078	NR	
Türkiye [40]	NR (*PTPN11*)	20 (Sanger)	20	-	p.Asn308Asp (25%/5)	80%/16	0.267	80%/16	0.402	30%/6	0.118

## 5. Conclusions

None of the symptoms were observed in all patients, confirming the wide variability observed among patients with NS. The majority of clinical data reported in our cohort aligns with data from other large studies published in the literature [25,43].

The most common features of NS in the investigated sample were facial dysmorphisms and cardiovascular system developmental abnormalities.

Frequent variants of the *PTPN11* gene were identified in Russian patients, accounting for more than 38% (c.922A>G p.Asn308Asp, c.417G>C p.Glu139Asp, c.1403C>T p.Thr468Met) of all cases with mutations in the *PTPN11* gene.

The application of NGS panels is justified in cases of Noonan syndrome and Noonan-like syndromes, which fall under the category of Rasopathies, as the extensive clinical variability even within a single gene does not allow for an accurate diagnosis without additional investigations.

A correctly diagnosed patient benefits from future management, increasing the potential for optimizing patient outcomes throughout their lifetime [44].

## Figures and Tables

**Table 1:** Shows the genetic diversity of variants in the *PTPN11* gene, identified in Russian patients. CM—classification in the HGMD Prof v. database (preferred), RCV—classification in the ClinVar database if the variant is not described in HGMD.

Position in cDNA	Exon	Position in the Protein	Domain	Number of Chromosomes	Variant Pathogenicity (ACMG Criteria for Previously Undescribed Variants)	HGMD ID	Frequency by GnomAD v.2.1.1	Frequency in RF (2600 Chromosomes)
c.124A>G	2	p.Thr42Ala	N-SH2	1	P	CM021125	-	-
c.172A>G	3	p.Asn58Asp	N-SH2	3	P	CM044250	-	-
c.174C>A	3	p.Asn58Lys	N-SH2	2	P	CM1619079	-	-
c.179G>C	3	p.Gly60Ala	N-SH2	4	P	CM021126	-	-
c.181G>A	3	p.Asp61Asn	N-SH2	1	P	CM021127	-	-
c.181G>C	3	p.Asp61His	N-SH2	1	P	CM101143	-	-
c.182A>G	3	p.Asp61Gly	N-SH2	5	P	CM013415	-	-
c.184T>G	3	p.Tyr62Asp	N-SH2	1	P	CM21128	-	-
c.188A>G	3	p.Tyr63Cys	N-SH2	4	P	CM013416	0.00001195	-
c.205G>C	3	p.Glu69Gln	N-SH2	1	P	CM030493	-	-
c.214G>T	3	p.Ala72Ser	N-SH2	1	P	CM013418	-	-
c.218C>T	3	p.Thr73Ile	N-SH2	1	P	CM021129	-	-
c.228G>C	3	p.Glu76Asp	N-SH2	1	P	CM013419	-	-
c.228G>T	3	p.Glu76Asp	N-SH2	3	P	CM060442	-	-
c.236A>G	3	p.Gln79Arg	N-SH2	3	P	CM013420	-	-
c.317A>C	3	p. Asp106Ala	C-SH2	2	P	CM021130	-	-
c.417G>C	4	p.Glu139Asp	C-SH2	11	P	CM021132	-	-
c.417G>T	4	p.Glu139Asp	C-SH2	1	P	CM021131	-	-
c.767A>G	7	p.Gln256Arg	PTP	1	P	CM030495	-	-
c.836A>G	7	p.Tyr279Cys	PTP	3	P	CM021133	-	-
c.844A>G	7	p.Ile282Val	PTP	2	P	CM013421	-	-
c.854T>C	7	p.Phe285Ser	PTP	1	P	CM021134	-	-
c.855T>G	7	p.Phe285Leu	PTP	1	P	CM073286	-	-
c.922A>G	8	p.Asn308Asp	PTP	23	P	CM013422	0.00001193	-
c.923A>G	8	p.Asn308Ser	PTP	5	P	CM021135	-	-
c.1391G>C	12	p.Gly464Ala	PTP	1	P	CM041070	-	-
c.1402A>C	12	p.Thr468Pro	PTP	1	P	CM074987	-	-
c.1403C>T	12	p.Thr468Met	PTP	6	P	CM021672	0.000003981	-
c.1471C>T	13	p.Pro491Ser	PTP	2	P	CM1711582	0.000003976	-
c.1472C>T	13	p.Pro491Leu	PTP	1	P	CM053389	-	-
c.1492C>T	13	p.Arg498Trp	PTP	1	P	CM041072	0.000003976	-
c.1493G>A	13	p.Arg498Gln	PTP	1	P (PM1, PM2, PM5, PP3, PP5)	RCV002471444.1	-	-
c.1502G>A	13	p.Arg501Lys	PTP	1	P	CM021137	-	-
c.1504T>A	13	p.Ser502Thr	PTP	1	P	CM022450	-	-
c.1507G>A	13	p.Gly503Arg	PTP	1	P	CM060440	-	-
c.1510A>G	13	p.Met504Val	PTP	3	P	CM013423	0.000003976	-
c.1528C>G	13	p.Gln510Glu	PTP	2	P	CM055503	-	-
c.1529A>C	13	p.Gln510Pro	PTP	1	P	CM043070	-	-
c.518G>T	4	p.Arg173Leu	C-SH2	1	VOUS (PM2, PP2, PP3)	-	0.00001591	-
c.1275C>G	11	p.Asp425Glu	PTP	1	VOUS (PM2, PP2, PP3) re-classified as LP (de novo)	-	-	-
c.1374C>G	11	p.His458Gln	PTP	1	VOUS (PM2, PP2, PP3) re-classified as LP (de novo)	-	-	-

## Data Availability

No new data were created or analyzed in this study. Data sharing is not applicable to this article.

## Supplementary Materials

The following supporting information can be downloaded at: https://www.mdpi.com/article/10.3390/genes15030345/s1, Table S1: phenotypes and variants of probands.
